# The Choice of a Health Resort

**Published:** 1888-07-07

**Authors:** 


### THE DOCTOR'S PULPIT.

### THE CHOICE OF A HEALTH RESORT.

THE time has again arrived when the industrious and the idle, the frivolous and the strenuous alike begin to think of their annual holiday. Some, indeed, have already sought the fresh breezes of Hastings and Bournemouth, Devonshire and Norfolk. There is no need to waste time in arguing the health value of one or two annual holidays. Regular readers are quite familiar with our views; and, we may hope, are convinced by our arguments. It is possible, as many people know by experience, at the end of a month's residence at the seaside, and after much expenditure of money, to be no better in health than before. An unwise choice has been made in the place, or a foolish use of the time of holiday. Few people, however, can afford to waste opportunities for improving health; and it is with the object of helping to a right decision that the present topic is selected for consideration.

There is a wide range of choice open to people of even moderate means. The country and the sea, the mountain and the plain, the British and the Continental health resorts, all have their merits and their eloquent advocates. Mrs. Ramsbotham has tried Scotland and Switzerland, Norway and even Ultima Thule, but she finds none of them equal to her beloved Margate? At Margate, fish or kidneys, bacon or hard-boiled eggs, chops with or chops without tomato sauce are relished with equal zest. If the good dame can eat nowhere else, she can always eat at Margate; and so, if her holiday be short and her purse incline to the lean and shrunken state, she gives little time to the weighing of the pros and cons, but decides at once for Margate. Undoubtedly, she is right. Experience is an excellent teacher when the pupil is blest with a reasonable modicum of common sense.

But, happily, everybody does not wish to go to Margate. It is not our purpose to tell them where they should go, but rather to help them to some general principles of guidance which may enable them to choose for themselves. Obviously one of the first things to be considered in the selection of a health resort is the selecting person. Is he young or old, gay or serious, well-developed or lean in the region of the calves? Does he like a promenade, a pier and a band; or does he prefer the deep heather and the stern-fronted mountains? Is he gouty and "short in the wind," or can he run a mile in six or seven minutes without being "breathed" or "turning a hair"? Let him sit down and settle points like these first of all, and then he will probably have narrowed his range of choice very greatly, and so have brought a decision much nearer than before.

Perhaps materfamilias is called upon to choose, and must exercise her choice with due regard to the necessities of "the children" or to the wishes of the "young ladies." In the latter event, the influencing motives may be so many and so contradictory that the good mother is almost driven to despair—she cannot please everybody. What is she to do? Choose a place which will suit her husband and herself, and let the young ladies make the best of it. A savage sentiment this, many young lady readers may be inclined to say! Not at all. When a Gordian knot cannot be untied, the practical person very often ends by cutting it.

Having decided as to the kind of person you are, what is the next point to be considered. This: What kind of place will be best for your health and give you most enjoyment? If you can make up your mind on these points, you have still further narrowed your radius of selection. Some people always get most benefit from the sea. They should incline to the sea. Others are never well by the sea, but improve in health at a quiet, inland place. An inland place by all means should have the preference for them. Some are content with neither sea nor inland spa, but long for the clear stimulating atmosphere and strenuous work of mountain climbing. For these Scotland, Switzerland, or parts of Devonshire, will probably be most suitable. Many people prefer a sea-trip to any other kind of holiday. Such trips are now easily available, and may be enjoyed in great variety. A patient of the writer's, two or three years ago, laid in a wonderful store of health by a three or four weeks' trip to Norway. For those who are even moderately good sailors it is difficult to imagine anything more enjoyable than a voyage to Norway or Shetland. In those far-off regions, too, there is not only a wonderful purity and stimulus in the atmosphere, but a refreshing simplicity of life and manners which surprises the worn-out man of business, and the woman weary and sick of conventionalities, into a new and altogether delightful childhood.

Men and women should not only consider the question of health when deciding upon a health resort, but also that of pleasure. A holiday enjoyed does twice as much good to the health as a holiday endured; whilst a delightful holiday is one of the greatest enemies to the doctor which he knows of. Nobody need be afraid to love innocent pleasure, or to seek it by all legitimate means. We are well aware, and the world knew very well long before Carlyle proclaimed the doctrine afresh, that "not pleasure but duty is the real end of life." But in order for a man or a woman to do the highest possible degree and kind of duty, it is necessary that work be occasionally interrupted by rest and pleasure. Let no person, therefore, who is resolved to spend life well, and only well, be the least afraid of giving way to the fullest possible enjoyment of innocent pleasure. In choosing a health resort the two leading objects to be kept in view should be health and brightness of mind.

It may have been noticed that most of the suggestions here made can be carried out without leaving these islands. The reasons are twofold: The first is, that persons of strictly limited leisure should not waste too much time in seeking far-away scenes for their holiday, but make it as long as possible by diminishing the time spent in mere travelling. The second, and less important one, is the patriotic reason of desiring to keep British wealth within British territories. There is no reason whatever why men of leisure and ample means should not travel on the Continent, or cross to America, or go yachting all round the world to their hearts' content. But we are not writing for these. Those to whom a holiday is an interval between strenuous bread-winning in the past, and equally strenuous bread-winning yet to come, should make the most of it for the two practical purposes of health and pleasure. For such a wise selection of place is a matter of first-rate importance, and if we have helped them, in ever so slight a degree to make such a selection, we shall be more than pleased.

